# Retrospective Analysis of Healthcare Resource Use, Treatment Patterns, and Treatment-related Events in Patients with Huntington’s Disease–associated Chorea Initiated on Tetrabenazine

**DOI:** 10.36469/9779

**Published:** 2018-01-08

**Authors:** Victor W. Sung, Ravi G. Iyer, Sanjay K. Gandhi, Victor Abler, Brian Davis, Debra E. Irwin, Karen E. Anderson

**Affiliations:** 1 University of Alabama at Birmingham, Department of Neurology, Division of Movement Disorders, Birmingham, AL; 2 Teva Pharmaceutical Industries, Frazer, PA; 3 Truven Health Analytics, an IBM Company, Cambridge, MA; 4 Truven Health Analytics, an IBM Company, Durham, NC; 5 Georgetown University, Washington, DC

**Keywords:** Huntington’s disease, chorea, tetrabenazine, healthcare resource, treatment-related events, retrospective study

## Abstract

**Background:**

Huntington’s disease (HD) is a multifaceted neurodegenerative disorder characterized by involuntary movements, specifically chorea, as well as behavioral and psychiatric disturbance, and cognitive dysfunction. Tetrabenazine was the first approved treatment for chorea, although tolerability concerns exist.

**Objectives:**

To characterize demographic and clinical characteristics of HD patients with chorea based on tetrabenazine use and examine treatment persistence with tetrabenazine in a real-world setting.

**Methods:**

Patients with a claim for HD-associated chorea (ICD-9-CM code 333.4) between 1/1/08 and 9/30/15 were selected from the MarketScan^®^ Commercial and Medicare Supplemental databases. The first diagnosis date during the study period was considered the index date, with ≥6 months of continuous medical and prescription coverage before and after the index date. Treatment persistence was defined as the number of days from initiation to discontinuation or end of follow-up period. Discontinuation was defined as a gap in therapy of ≥60 days.

**Results:**

1644 patients met selection criteria (mean age ± standard deviation: 54.5 ± 15.5), of which 151 (9.2%) were treated with tetrabenazine during the study period. The average (median) daily dose of tetrabenazine during the treatment period was 45.5 (42.3) mg/day. A total of 41.8% (59/141) of HD patients who initiated tetrabenazine experienced a ≥60-day gap in tetrabenazine therapy, with a median time to discontinuation of 293.5 days. During the 6-month post-index period after HD diagnosis, HD patients incurred higher all-cause healthcare costs ($20 204) vs the 6-month pre-index period ($6057), driven by higher hospitalization and pharmacy costs.

**Conclusions:**

A small percentage of HD patients with chorea were treated with tetrabenazine and discontinuation rates were high among those receiving treatment, with a median time to discontinuation of 9 months.

## Background

Huntington’s disease (HD) is a hereditary, progressive neurodegenerative disorder characterized by motor dysfunction, cognitive impairment, and behavioral-emotional symptoms.^1^–^3^ The most common motor symptom of HD is chorea, which affects about 90% of HD patients at some point during the course of their disease.^4^ In addition to being one of the most visible features of HD, chorea can also increase the risk of injury and interfere with daily functioning.^4^,^5^

Vesicular monoamine transporter 2 (VMAT2) is responsible for the storage and release of dopamine from synaptic vesicles in the brain.^6^,^7^ Tetrabenazine, a VMAT2 inhibitor that modulates dopamine, was approved by the US Food and Drug Administration (FDA) as a treatment option for chorea associated with HD in 2008.^8^,^9^ In a randomized, double-blind, placebo-controlled study of tetrabenazine in HD patients (TETRA-HD), tetrabenazine significantly improved chorea, but its use was associated with an increased risk of neurologic and psychiatric adverse events (AEs), including drowsiness/somnolence, insomnia, fatigue, fall, depression, agitation, and anxiety.^10^ These neuropsychiatric AEs, likely due to high peak concentrations and plasma fluctuations, cause tolerability concerns that may limit the real-world use of tetrabenazine.^10^,^11^

There is a lack of real-world studies characterizing tetrabenazine use in patients with HD. The primary objective of this study was to characterize demographic and clinical characteristics of HD patients with chorea based on tetrabenazine use utilizing real-world data from the MarketScan^®^ Commercial and Medicare Supplemental Databases. We also analyzed treatment persistence and dosing with tetrabenazine. We sought to better understand prescription patterns of tetrabenazine and examine relevant neurologic and psychiatric comorbidities and healthcare resource use and costs in HD patients with chorea who were initiated on tetrabenazine.

## Methods

### Data Sources

This retrospective analysis was conducted using administrative medical and pharmacy claims data from the Truven MarketScan^®^ Commercial Claims and Encounters (Commercial) Database for the period between January 1, 2008 and September 30, 2015. This database contains complete longitudinal records of inpatient and outpatient services, and prescription drug claims of more than 45 million employees and their dependents, covered under a variety of fee-for-service, fully capitated, and partially capitated health plans across all geographic regions of the United States. All study data were de-identified and fully compliant with Health Insurance Portability and Accountability Act (HIPAA) of 1996. This study used only deidentified patient records and did not involve the collection, use, or transmittal of individually identifiable data; therefore, Institutional Review Board approval to conduct this study was not necessary.

### Study Population

Adult patients diagnosed with HD chorea (aged 18 years and older) initiated on tetrabenazine (i.e. no tetrabenazine use in the prior 6 months) with at least one non-diagnostic claim for HD chorea (ICD-9-CM code 333.4) between January 1, 2008 and September 30, 2015 were identified. All patients were required to be continuously enrolled with medical and pharmacy benefits for at least 6 months before and after the diagnosis index date (first date of HD diagnosis). Patients were excluded from the study if they had evidence of an HD diagnosis or tetrabenazine use at any point in time while they were continuously enrolled prior to the diagnosis index date. HD patients who initiated tetrabenazine after the diagnosis index date were included in the tetrabenazine treatment group. For treatment-related outcomes, the treatment index date was the date of the first tetrabenazine claim in the study period. We examined the outcomes for 6 months and for a variable follow-up period.

### Outcomes

All-cause healthcare utilization and costs, reported by type of service (inpatient, outpatient, and pharmaceutical), were measured during the pre-index and post-index periods. Healthcare costs were based on paid amounts of adjudicated claims, including insurer and health plan payments as well as patient cost-sharing in the form of co-payment, deductible, and co-insurance. Cost for services provided under capitated arrangements were estimated using payment proxies that were computed based on paid claims at the procedure level using the MarketScan Commercial and Medicare Supplemental Databases. All dollar estimates were inflated to 2015 dollars using the Medical Care Component of the Consumer Price Index (CPI).

Treatment patterns were measured during the entire treatment episode, occurring during the variable-length follow-up period. Treatment discontinuation was defined by a gap in days’ supply of tetrabenazine of at least 60 days. Time to first treatment discontinuation was the number of days from the index date through the last day of supply of the index medication prior to treatment discontinuation. Sensitivity analyses were conducted to assess the discontinuation for both 30- and 90-day gaps.

Treatment persistence for tetrabenazine treatment was defined as the number of days from initiation to discontinuation or end of follow-up period, whichever occurred first. A binary variable was created to indicate whether the patient remained persistent throughout the post-index period and continuous variables were created to capture time to non-persistence.

Treatment compliance was measured during a defined 6-month follow-up period after initiation of drug. In order to be assessed, all patients were required to have continuous enrollment in medical and pharmacy coverage during the entire length of follow-up defined, and have at least two prescription claims for tetrabenazine. Compliance was measured using proportion of days covered (PDC).

The average daily dose of tetrabenazine was calculated for each prescription refill during the first 12 prescription claims treatment episode using the quantity of pills, days’ supply of drug, and drug strength to evaluate whether or not there were dose changes occurring over the course of the treatment episode.

Other outcomes included new incidence of relevant neuropsychiatric comorbidities/diagnoses and use of concomitant medications associated with tetrabenazine treatment. The incidence of comorbidities/diagnoses (eg, new diagnoses of depression, Parkinson’s disease symptoms, anxiety) and pharmacy claims for antidepressants, anxiolytics, or anti-drowsiness medications were examined over the variable-length follow-up period.

### Statistical Analysis

All study variables, including demographic and clinical characteristics for the overall sample, were summarized descriptively. Statistical tests of significance were performed for differences between the preand post-treatment index periods for healthcare costs. Chi-square tests were used to evaluate the statistical significance of differences for categorical variables. To evaluate the statistical significance of differences for normally distributed continuous variables, t-tests were used. An *a priori* P value of <0.05 was set as the threshold for statistical significance. Kaplan–Meier curves were plotted to examine the discontinuation rate and time to discontinuation, including the sensitivity analysis.

## Results

### Study Population

**Figure 1:**
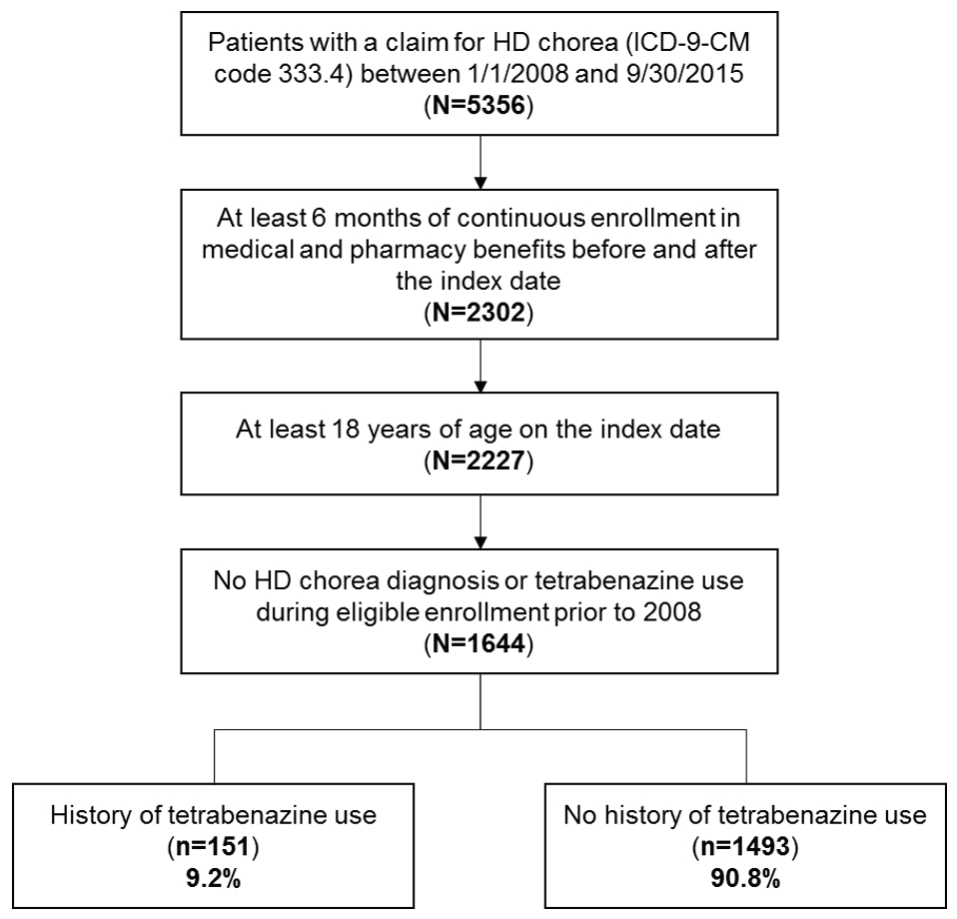
Study Sample Selection Flowchart HD: Huntington’s disease

Among 5356 patients identified with a claim for HD chorea between 1/1/2008 and 9/30/2015, 1644 patients met the selection criteria. Of the 1644 HD chorea patients, 151 (9.2%) had a history of tetrabenazine use during the follow-up period (Figure 1). Baseline demographic characteristics of these patients are shown in Table 1. Median time from initial diagnosis of HD chorea during the study period (diagnosis index date) to tetrabenazine claim was 158.5 days. The mean (standard deviation, SD) age of the 151 patients included in the study was 55.5 (12.5) years and 50.3% were female. The mean (SD) follow-up period was 369 (414) days after the initiation of tetrabenazine. Of the 151 patients receiving tetrabenazine, 141 patients had sufficient data to allow the evaluation of tetrabenazine use pattern outcomes (persistence, compliance, average dose). All 151 patients were included in the analysis of healthcare costs and resource use.

Out of 5356 patients with a claim for HD chorea between 1/1/2008 and 9/30/2015, 1644 patients had at least 6 months of continuous enrollment in medical and pharmacy benefits before and after the index date, were at least 18 years of age on the index date, and had no HD chorea diagnosis or tetrabenazine use during eligible enrollment prior to 2008, and were included in this study. Of these eligible patients, 151 (9.2%) had a history of tetrabenazine use and 1493 (90.8%) had no history of tetrabenazine use.

### Healthcare Resource Utilization and Costs Before and After HD Diagnosis

Overall, a higher proportion of HD patients had evidence of healthcare resource use in the 6-month period post HD diagnosis compared to the 6-month period of time prior to HD diagnosis. The average length of stay and inpatient days were higher in the 6-month post-index period, and HD patients incurred higher allcause healthcare costs in the 6-month post-index period vs the 6 month pre-index period ($20 204 vs $6057, P<0.001) (Table 2). This increase was driven by higher hospitalization costs ($6579 vs $2961, P=0.083) and higher pharmacy costs ($9263 vs $916, P<0.001) (Table 2).

### Dosing

**Figure 2:**
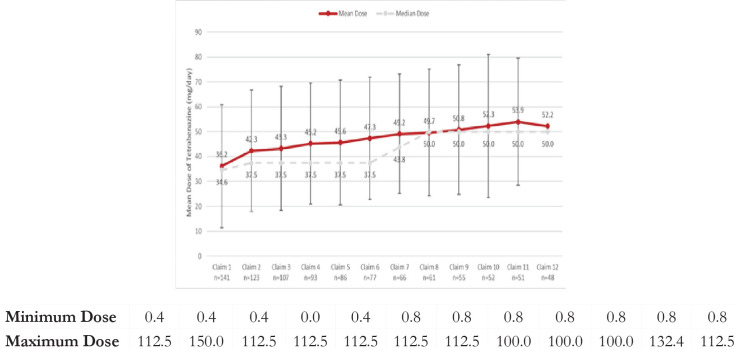
Mean and Median Dose for Claims 1 Through 12 Mean and median dose for claims 1 through 12 show a gradual increase in the dose from previous fill to the next fill. The average daily dose only increased to 50 mg/day by the ninth refill. Error bars indicate standard deviation.

There was a mean (median) of 6.8 (6.0) prescription claims for tetrabenazine during the treatment episode. There was a gradual increase in the dose, starting with an average (median) daily dose of 36.2 (34.6) mg/day for the first tetrabenazine fill, and ending with average (median) daily dose of 50.6 (50.0) mg/day for the last tetrabenazine fill, up to the 12th prescription fill. The mean (median) daily dose of tetrabenazine during the treatment period was 45.5 (42.3) mg/day. The majority of tetrabenazine prescriptions (706/960, 73.5%) were ≤50 mg/day. There was a gradual increase in the dose from previous fill to the next fill; however the average daily dose only increased to 50 mg/day by the twelfth refill (Figure 2).

### Persistence and Compliance

**Figure 3:**
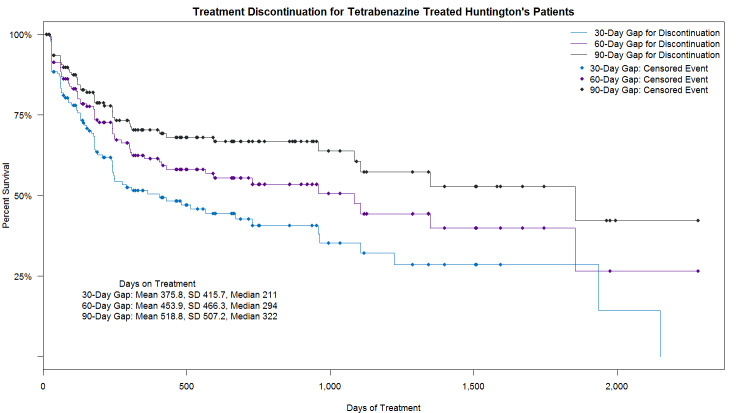
Kaplan–Meier Curve: Time to Tetrabenazine Discontinuation Mean and median time to discontinuation for HD chorea patients receiving tetrabenazine treatment for 30-, 60-, and 90-day gaps. HD: Huntington’s disease; SD: standard deviation

Approximately 58.2% (82/141) of patients remained persistent on therapy over the entire follow-up period. A total of 41.8% (59/141) of HD chorea patients who initiated tetrabenazine discontinued tetrabenazine therapy (defined as a ≥60-day gap), with a median time to discontinuation of 293.5 days (Figure 3). Of the patients who discontinued tetrabenazine, 40.7% (24/59) re-initiated treatment, with a mean (SD) days to reinitiation of 133.2 (100.8) days. Within approximately 6 months, 25% of HD chorea patients discontinued tetrabenazine, based on a 60-day gap. The mean (SD) PDC during the 6-month follow-up period was 0.83 (0.26) and 72.7% of patients had a PDC >0.8.

### Incidence of Comorbidities/Co-diagnoses and Use of Concomitant Medications

Following the initiation of tetrabenazine treatment, 14.9% of the 141 patients developed a new onset of depression or had a new prescription for an antidepressant, and 12.8% of the population developed anxiety or had a new prescription for anxiolytics (Table 3). Diagnoses for drowsiness or anti-drowsiness prescriptions were newly indicated in 14.2% of tetrabenazine patients (Table 3).

## Discussion

This study utilized a large administrative healthcare database, MarketScan^®^ Commercial and Medicare Supplemental, to characterize tetrabenazine use among patients with HD chorea. These databases provided in-depth, cross-sectional, and longitudinal views of healthcare practices in the US and detailed information from inpatient and outpatient settings. This study demonstrates the high economic burden in this HD patient population and is consistent with findings from other healthcare resource use and cost studies in the United States and United Kingdom.^12^,^13^

Real-world data suggest that a small percentage of HD patients were prescribed tetrabenazine, and those who were prescribed tetrabenazine were on suboptimal doses and did not remain on the drug for extended periods of time. Despite tetrabenazine being the only FDA-approved for the treatment of HD chorea during the time of this study (2008–2015),^8^,^14^ only about 9.2% of patients diagnosed with HD chorea were prescribed tetrabenazine. Other database analyses have reported similar low rates of tetrabenazine use, ranging from 8.7 to 14.4%.^15^,^16^ In a survey of HD patients and caregivers, nearly 40% of respondents noted being unaware of or not utilizing any medication to treat chorea, with just 22.9% of respondents taking tetrabenazine to treat HD chorea.^17^ These data may reflect doctors’ hesitation to prescribe tetrabenazine due to concerns with safety and tolerability.^10^

Although there is no maximum optimal/allowable dose for patients initiated on tetrabenazine, a majority of the patients who completed the TETRA-HD study received a dosage above 50 mg/day in the maintenance phase.^10^ Tetrabenazine dosing is dependent on the CYP2D6 metabolizing status of the patient. For intermediate and extensive CYP2D6 metabolizers, the maximum recommended daily dose is 100 mg and the maximum recommended single dose is 37.5 mg. For poor CYP2D6 metabolizers, the maximum recommended single dose is 25 mg, and the recommended daily dose should not exceed a maximum of 50 mg.^8^

The results from the current analysis revealed that over 73% of HD of prescriptions were dispensed at the suboptimal doses of ≤50 mg/day. In addition, the average dose of tetrabenazine was 45.5 mg/day over the initial 12 prescriptions. Moreover, over 40% of patients received a dose of ≤50 mg/day for their last claim. These findings are consistent with other claims data and electronic medical records data analyses, which showed that HD chorea patients receive an average daily dose of 41–58 mg of tetrabenazine in real-world clinical settings.^15^,^16^ This study does not make a direct comparison to the results in the pivotal trial; however, the mean dosing seen in the real-world setting is lower than what was attained in the clinical trial (75 mg/day).^10^

Persistence to tetrabenazine treatment was low, with more than 40% of patients discontinuing within 1 year, 59% of whom remained off treatment. The rate of discontinuation in the real world is consistent with what has been seen in clinical trials. About 54% of the patients reduced the dose or discontinue therapy in TETRA-HD due to side effects.^10^ These low rates of tetrabenazine persistence might be due to the AEs associated with the drug, or dissatisfaction with efficacy.^10^ In a previous survey of HD patients and caregivers, about 50% of respondents reported that treatments were “somewhat,” “not very well,” or “not at all” effective in managing chorea.^17^ Studies have shown that adverse drug events reduce drug adherence, and have identified strategies to improve medication adherence, including patient education and counseling, involving family members, and adequate healthcare professional training.^18^–^21^ Further research is needed to better understand the reasons for low treatment initiation, suboptimal dosing, and high discontinuation, as these could not be investigated in the current study due to limitations of claims data.

There are several limitations of this study. Some of the comorbidities examined (e.g. depression, anxiety) are common in HD patients, and we cannot determine from these data whether new onset of depression after tetrabenazine initiation was causally due to tetrabenazine treatment or the underlying HD disease process. To better determine the link between tetrabenazine treatment, comorbidities, and discontinuation, future studies should look at patient data in which attribution of tolerability effects and AEs are recorded over the course of HD diagnosis and tetrabenazine treatment. Another limitation is the inability to determine patients CYP2D6 metabolizing status in claims data. Patients who are poor CYP2D6 metabolizers may have received a lower-than-average dose.

These data highlight the need for efficacious, tolerable, and continuous treatments for patients with HD that do not exacerbate psychiatric comorbidities associated with HD. Deutetrabenazine, which was approved in 2017 for the treatment of HD chorea,^14^ may improve tolerability in patients with HD by reducing AEs associated with peak concentration.^11^,^22^,^23^ An indirect comparison of tetrabenazine and deutetrabenazine (the TETRA-HD and First-HD trials, respectively) found that deutetrabenazine has a significantly lower risk for moderate to severe AEs compared with tetrabenazine.^24^ Furthermore, an open-label study has demonstrated that patients with HD can safely be converted from tetrabenazine three times daily to deutetrabenazine twice-daily with good adherence.^25^

## Conclusions

HD is a multifaceted disease that requires comprehensive clinical care and services for individuals and families affected by HD. Tetrabenazine is an important and efficacious treatment for HD-associated chorea; however, our data highlight potential barriers to its use and emphasize the continued need for better, safer, and more-tolerable treatments with personalized support for patients with HD.

## Figures and Tables

**Table 1:** Baseline Demographic and 6 Month Pre-diagnosis Clinical Characteristics of HD Chorea Patients who Received Tetrabenazine (N=151)

	HD Chorea Patients who Received Tetrabenazine
**Demographic Characteristics**	
Age, mean (SD)	55.5 (12.5)
Female, n (%)	76 (50.3)
**Insurance Plan Type, n (%)**	
Commercial	119 (78.8)
Medicare	32 (21.2)
**HD Severity in Pre-Index Period, n (%)**^a^	
Early stage	139 (92.1)
Middle state	7 (4.6)
Late stage	5 (3.3)
**Deyo-Charlson Comorbidity Index score in Pre-index Period, mean**	(SD) 0.3 (0.9)

a HD severity measure was adapted from: Divino et al. 2013.12

HD: Huntington’s disease; SD: standard deviation.

**Table 2:** Healthcare Resource Utilization and Costs Pre- and Post-diagnosis of HD

	HD Patients With Tetrabenazine Use (n=151)				Δ Pre–Post
	6-Month Pre-diagnosis		6-Month Post-diagnosis		
	N/Mean	%/SD	N/Mean	%/SD	
**All-cause Utilization and Expenditures**					
Number of Patients with (n, %)					
Inpatient admission	7	4.6	19	12.6	7.9*
Emergency room visit	25	16.6	32	21.2	4.6
Outpatient visit	123	81.5	151	100.0	18.5**
Office visit	117	77.5	148	98.0	20.5**
**Utilization (Mean, SD)**					
Inpatient admissions	0.07	0.45	0.20	0.63	0.13*
Inpatient days	0.48	4.51	2.65	16.81	2.17
Average length of stay	0.19	1.08	1.09	5.21	0.91*
Emergency room visits	0.27	0.77	0.41	1.13	0.14
Outpatient visit	18.19	24.16	26.71	29.75	8.52**
Office visit	3.34	3.71	5.02	3.27	1.68**
**Expenditures (Mean, SD), $**					
Inpatient admissions	2961	31 637	6579	33 930	3619
Emergency room	191	764	322	1189	132
Outpatient visit	2180	4010	4362	6828	2180**
Office visit	404	433	642	405	238**
Outpatient pharmacy	916	1303	9263	12 804	8333**
**Total All-Cause Healthcare Costs**	6057	32 774	20 204	41 264	14 132**

HD: Huntington’s disease; SD: standard deviation

* P<0.05;

** P<0.001

**Table 3:** Newly Diagnosed Neuropsychiatric Events in HD Patients Who Received Tetrabenazine During the Variable-Length Post-treatment Period (N=141)

Treatment-Related Events	Variable-Length Treatment Episode^1^, Incident Events^2^	
Comorbid Conditions	N	%
Bipolar disorder	6	4.3
Dysarthria	1	0.7
Fall	9	6.4
Fatigue	16	11.3
Insomnia	16	11.3
Parkinson’s Disease	9	6.4
Suicide and suicide ideology	2	1.4
Diagnosis for depression and/or prescription for antidepressants	21	14.9
Diagnosis for anxiety and/or prescription for anxiolytics	18	12.8
Diagnosis for sedation/somnolence and/or prescription for anti-drowsiness medications	20	14.2

HD: Huntington’s disease;

1 Variable-length post-treatment period assessed conditions and medications that occurred between the start and end of tetrabenazine treatment;

2 Events were considered incident if they had not been observed during the 6-month pre-treatment period.
